# Pre- and post-weaning scours in southeastern Australia: A survey of 22 commercial pig herds and characterisation of *Escherichia coli* isolates

**DOI:** 10.1371/journal.pone.0172528

**Published:** 2017-03-08

**Authors:** Lechelle K. Van Breda, Om P. Dhungyel, Andrew N. Ginn, Jonathan R. Iredell, Michael P. Ward

**Affiliations:** 1 The University of Sydney, Faculty of Veterinary Science, Camden, Australia; 2 Centre for Infectious Diseases and Microbiology, The Westmead Millennium Institute for Medical Research, The University of Sydney, Westmead Hospital, Westmead, Australia; 3 Antimicrobial Resistance Reference Laboratory, Centre for Infectious Diseases and Microbiology Laboratory Services, Pathology West, Westmead, Australia; University of Minnesota, UNITED STATES

## Abstract

Diarrhoeal diseases in piglets caused by *Escherichia coli* are responsible for substantial losses each year in the Australian pig industry. A cross-sectional survey was conducted (September 2013–May 2014) across 22 commercial pig herds located in southeastern Australia: NSW (n = 9); VIC (n = 10); and SA (n = 3), to estimate the prevalence of *E*. *coli* associated diarrhoea in pre- and post-weaned piglets and to identify key risk factors associated with *E*. *coli* disease. A questionnaire on management and husbandry practices was included. Faecal samples (n = 50 from each herd) were tested for the presence of β-haemolytic *E*. *coli*. Species level identification was confirmed by matrix-assisted laser desorption / ionisation time-of-flight mass spectrometry (MALDI-TOF/MS). ETEC virulence and enterotoxin genes (F4, F5, F6, F18, F41, STa, STb and LT) were screened for by multiplex PCR. This study assessed 60 potential risk factors for *E*. *coli* disease in post-weaned piglets, with 2 key factors–recent disease events and the presence of bedding, statistically associated with the presence of post-weaning scours. The prevalence of diarrhea in pre-weaned pens was 17% (16/93), compared with 24% (24/102) in post-weaned pens. The most prevalent β-haemolytic ETEC genes were F18 (32%) and STb (32%) but isolates were more likely to contain F4:STb (11 of 22 herds, 23%), than F18:STb (5 of 22 herds, 6%). These findings indicate that recent disease events that have occurred within the last 12 months, and by the use of bedding or not maintaining fresh bedding can have significant impacts on piglet diarrhoea.

## Introduction

Post-weaning diarrhoea caused by *Escherichia coli* remains a major disease that limits production in pig farms worldwide. Pathogenic *E*. *coli*–in particular enterotoxigenic *E*. *coli* (ETEC) may express fimbrial adhesins (F4 (K88), F5 (K99), F6 (987P), F18 and F41) [1] and enterotoxins, (heat-stable, STa and STb, Stx2e and heat-labile, LT) [2], that are associated with more severe disease in piglets [3, 4]. ETEC colonise the lower intestine via these fimbrial adhesins and the production of enterotoxins results in diarrhoea ("scouring") that can lead to death if left untreated.

Australian pig herds regularly experience outbreaks of pre- and post-weaning diarrhoea attributed to *E*. *coli*, resulting in reduced growth rates, high medication costs and high morbidity and mortality [4]. Madec and Josse [5] used an ecopathological approach to identify 10 key ‘risk factors’ from among 515 environment factors measured in a study of diarrhoea incidence in 89 French pig herds. A longitudinal study on Western Australian pig herds [6, 7] assessing a comprehensive French risk factor protocol developed by Madec [8], failed to identify reliable risk predictors of post-weaning diarrhoea. It has been estimated that piglet scours cost the Australian pig industry more than $7 million each year [9] but there are scant information regarding the epidemiology of *E*. *coli* disease in modern pig production systems in Australia, or of management practices that contribute to or ameliorate disease outbreaks.

Australian pig herd management strategies have evolved substantially over the last 20 years with weaners now commonly raised on deep litter (Eco shelters), and in large groups of >200 piglets per shelter. Other important changes include; sow group housing, phasing out of sow stalls, improvements in diet and nutrition, improvements in disease management through vaccines and strict biosecurity procedures, increases in productivity and an increase in the number of free range herds. It is therefore important to determine whether previously identified risk factors for post-weaning scours remain important and whether other factors need to be considered. Current estimates of the prevalence of pre- and post-weaned piglet scours in commercial pig farms in the major pig-raising area of southeastern Australia are lacking. Published studies on management practices and other factors that may contribute to outbreaks of post-weaning scours are also needed. The aim of the current study was to estimate the within-herd prevalence of pre- and post-weaning scours and to report the management practices–including current disease and biosecurity, environmental factors and reproductive practices, housing and herd management and their association with post-weaning scours in 22 representative pig herds across southeastern Australia.

## Methods

### Study design

The study was designed as a cross-sectional survey to estimate point prevalence of *E*. *coli* disease in piglets at the pen and herd levels. A total sample size of 22 herds was chosen, based on the available funding and ability to visit and sample all herds over a large geographical area during a relatively short time period. All animal sampling procedures and interactions were carried out in strict accordance with the recommendations made by The University of Sydney Animal Ethics Committee. The protocol was approved by The University of Sydney Animal Ethics Committee (Approval number: N00/7-2013/3/6002).

Farms were identified via swine veterinarians and in addition, farms were recruited at the 2013 Bendigo Pig Fair, Victoria and by contacting farmers directly via industry membership. A preliminary questionnaire was conducted over the telephone to determine the owner/manager's willingness to participate in the study and the suitability of their herd (S1 File). For example, herds with less than 40 sows were not included because the number of piglets available for sampling would not meet the required sample size for inclusion in the study. All questions in the questionnaires were carried out in strict accordance with the recommendations made by The University of Sydney Human Ethics Committee. The protocol was approved by The University of Sydney Human Ethics Committee (Approval number: 2013/827).

### Questionnaire

A semi-structured questionnaire was conducted once on-site face-to-face with the owner, manager or the leading farm hand at each farm (S2 File). It was used to gather information on current disease and biosecurity, environmental factors and reproductive practices, housing and herd management. The questionnaire took approximately 20 minutes to complete. Of the 40 questions, nine questions were structured as closed (answers limited to yes, no or occasionally), eleven were semi-closed (for example, age at weaning or number of pigs per pen) and 20 were open (for example, occurrence of recent diseases or feed company suppliers) [10, 11]. Two questions required observational and diagrammatical records to be made (as photography was not permitted), to document design and size of the pre- and post-weaning facilities and assist in developing a visual representation of the size, shape and design of the farrowing crate/pen and the weaner environment.

### Sampling

A cross-sectional survey was conducted between September 2013 and May 2014 of 22 commercial herds located in southeastern Australia (Victoria, n = 10; New South Wales, n = 9; and South Australia, n = 3). Herds were classified based on location (state: VIC, NSW, SA) and size (small/medium 45–499 sows, large ≥500 sows).

The aim was to collect faecal samples from piglets in the age group of one week prior to weaning and then the piglets that had been most recently weaned (3–6 weeks of age) on the day of the herd visit. In collecting samples from a pen, the aim was to represent the entire pen environment in terms of both scour and non-scour samples. Faecal samples were collected from each herd (10 samples from pre-weaned and 40 samples from post-weaned piglet pens depending on the size of the pen). If there were 20 or less piglets in a pen, then five samples were collected from the pen. If there were >20 piglets in a pen, then 10 samples were collected per pen. A total of 195 pens (93 pre- and 102 post-weaned) were sampled across 22 pig herds.

Faecal samples (approximately 2 g) from individual faecal pats at separate collection points within the pen were transferred into sterile 5 mL tubes. During collection, the samples were categorised as clinical (scours) or non-clinical (normal) based on a faecal consistency score developed by Pedersen and Toft [12] with slight modifications; 1 = dry and hard, 2 = firm and hard, 3 = firm and shaped (normal), 4 = soft and wet, and 5 = watery/liquid (diarrhoea). Diarrhoea positive pens were categorised based on the isolation of one or more diarrhoea samples. Samples were kept on ice until they could be stored at 4°C in the laboratory, for up to a week.

Samples were brought to room temperature (23°C) and a sterile 10 μL loop (Sarstedt) was used to homogenise the faecal sample and then it was streaked onto a Sheep Blood Agar plate (SBA) and incubated overnight aerobically at 37°C. The cultures were screened for β-haemolytic colonies (a cleared zone) on the SBA. Suspected β-haemolytic *E*. *coli* colonies were subcultured onto CHROMagar Oientation (CHROMagar^™^). Species identification was confirmed by matrix-assisted laser desorption / ionisation time-of-flight mass spectrometry (MALDI-TOF/MS) (Microflex LT MALDI BioTyper; Bruker Biosciences, Preston, VIC, Australia). Single pure colonies were cultured by transferring into 3 mL of sterile Buffered Peptone Water (BPW) (BD Difco^™^) and incubated aerobically overnight at 37°C. Then 500 μL of enriched culture was dispensed into sterile 1.5 mL screw capped tubes (Scientific Specialties) and stored at 4°C for up to a week until DNA extraction. To the remaining 2.5 mL of enriched culture, 80% glycerol was added and mixed well, before being stored at –80°C for further use.

Sterile BPW (5 mL) was added to each of the remainder samples, mixed well and incubated aerobically overnight at 37°C for enrichment. To each of these enriched faecal samples, 80% glycerol was added and mixed well prior to long term storage at –80°C.

### Molecular biology

Lysates were prepared by vortexing the samples and boiling at 100°C for 10 min. Samples were then centrifuged (2 min at 1000 x *g*) and 200 μL of supernatant transferred to a new tube and again centrifuged (10 min at 16,000 x *g*). The supernatant (200 μL) was used as a template in multiplex PCR before being stored at –20°C in 1.5 mL screw top tubes (Scientific Specialties).

One haemolytic *E*. *coli* isolate from each sample was tested for the presence of virulence genes (F4, F5, F6, F18 and F41) and enterotoxin genes (STa (*estA*), STb (*estB*), Stx2e (*stx2e*) and LT (*elt*)) by multiplex polymerase chain reaction (PCR) [13–15]. DNA from pure cultures of the isolates was amplified by a multiplex PCR kit protocol (QIAGEN) with slight modifications (Table 1).

**Table 1:** Primers used to detect *E*. *coli* virulence and toxin genes in a survey of southeastern Australian pig herds.

Virulence attributes	Primer sequence (5’– 3’)		Amplified product (bp)
	Forward	Reverse	
F4 ^a^	GGTGATTTCAATGGTTCG	ATTGCTACGTTCAGCGGAGCG	764
F5 ^a^	TGGGACTACCAATGCTTCTG	TATCCACCATTAGACGGAGC	450
F6 ^a^	TCTGCTCTTAAAGCTACTGG	AACTCCACCGTTTGTATCAG	333
F18 ^b^	GTGAAAAGACTAGTGTTTATTTC	CTTGTAAGTAACCGCGTAAGC	510
F41 ^a^	GAGGGACTTTCATCTTTTAG	AGTCCATTCCATTTATAGGC	431
STa ^a^	TCTTTCCCCTCTTTTAGTCAG	ACAGGCAGGATTACAACAAAG	166
STb ^a^	ATCGCATTTCTTCTTGCATC	GGGCGCCAAAGCATGCTCC	172
LT ^b^	GGCGACAGATTATACCGTGC	CCGAATTCTGTTATATATGTC	696
Stx2e ^c^	AATAGTATACGGACAGCGAT	TCTGACATTCTGGTTGACTC	733

^a^ [14],

^b^ [13],

^c^ [15]

A reaction volume of 20 μL was used for PCR, containing 2 μL of DNA template, 0.2 mM of each primer (1 μL), 2x QIAGEN Multiplex PCR Master mix (6 μL), 5x Q–Solution (3 μL) and 3–5 μL of nuclease-free water (QIAGEN Multiplex PCR Kit protocol). PCR conditions were 95°C denaturation for 15 min, 30 cycles of 94°C denaturation for 1 min, 53–58°C annealing for 1 min, 72°C extension for 1 min, and a final extension step of 72°C for 7 min. Amplified DNA was separated on 2% 1 x TBE agarose (Bioline) gel and measured with the molecular size marker (1013-bp ladder) 0.5 μg/μL of pUC Mix Marker, 8 (Fermentas), stained with ethidium bromide 0.5 μg/mL. DNA bands were observed under UV light using the Gel Doc XR Fluorescent Imaging System (Bio-Rad, Hercules, CA).

### Statistical analysis

The data are collated in Excel (Microsoft, PC/Windows XP, 2010, USA) and descriptive statistical analysis was performed using Genstat 16^th^ Edition (2000–2015 VSN International Ltd). Proportions were compared using the Mann-Whitney U-test or Kruksal-Wallis one-way ANOVA and all risk factors were categorised and analysed by Fisher’s exact test. Analysis of multiple predictors of pen-level post-weaning scours was performed using step-wise forwards logistic regression considering only those factors significant p<0.2 in univariate analysis and retaining only factors significant at p≤0.05 in the final model. Since pens were nested within a herd, a random effects term was included to account for lack of independence between pens within herd.

## Results

### Farm survey

Farms were classified into groups based on the state in which they were located. The mean herd size was 721 sows, with a wide range (from 45 to 20,000 sows per farm) so for the purpose of this study we classified farms into two groups, small/medium (45–499 sows) and large producers (≥500).

The questionnaire data are categorised. Demographics, disease occurrence and biosecurity; weaner environment; herd reproductive strategies and weaner housing and husbandry practices of the surveyed herds are summarised in Tables 2 and 3 (S3 File). Up to 10% of the data are missing due to some farmers declining to answer all questions.

**Table 2:** Disease, biosecurity, environmental and reproductive management practices in 22 pig herds across southeastern Australia (Victoria, VIC; New South Wales, NSW; South Australia, SA) included in a survey of pre- and post-weaning scours.

		State			Farm size (sows)	
Variable	Category	VIC	NSW	SA	≤499	≥500
Farms		58	110	27	123	72
Scours during suckling	Yes / sometimes	46	98	27	103	68
	No	12	12	0	20	4
Scours during post-weaning	Yes / sometimes	30	84	27	97	44
	No	28	26	0	26	28
*E*. *coli* vaccine for sows	Yes	35	87	17	78	61
	No	14	23	10	37	10
Vaccination for sows	Yes	49	98	27	103	71
	No	0	12	0	12	0
*E*. *coli* vaccine for suckers	Yes	0	21	0	0	21
	No	58	89	27	123	51
Vaccination for suckers	Yes	17	38	21	33	38
	No	41	72	6	90	34
*E*. *coli* vaccine for weaners	Yes	0	15	0	15	0
	No	50	84	27	89	72
Vaccination for weaners	Yes	39	28	16	57	26
	No	11	71	11	47	46
Recent diseases (within the last 12 months)	Yes	30	65	0	57	38
	No	28	45	27	66	34
Frequency weaner pens cleaned	Frequently ≤9 weeks	23	64	0	47	40
	End of each batch ≥10 weeks	26	27	27	66	14
Disinfectant and pressure hosing used to clean weaner pens	Yes	33	29	10	47	25
	No	1	12	0	12	0
Size of weaner pen	Small (1–15 piglets)	0	40	0	40	0
	Medium (16–199)	32	51	16	34	55
	Large (≥200)	26	19	11	49	17
Bedding in suckers pen	Yes	4	11	0	15	0
	No	53	99	27	108	71
Bedding in weaner pen	Yes	18	27	17	57	5
	No	40	83	10	66	67
Type of bedding	Straw	6	0	6	8	4
	Straw and another type of bedding	12	0	11	22	1
	Other	0	27	0	27	0
Housing type sampled	Conventional sheds	40	99	10	82	67
	Conventional sheds and Eco shelters	14	11	17	37	5
	Outdoor/free range	4	0	0	4	0
Flooring	All inorganic	52	59	21	60	72
	Organic	6	51	0	57	0
Shed ventilation	Fair	56	87	27	98	72
	Poor	2	23	0	25	0
Farrowing system	All in/all out^a^	50	94	27	99	72
	Continuous flow	8	16	0	24	0
Average weaning age	≤21 days	31	11	21	37	26
	≥22 days	27	99	6	86	46
Weaner groups housed in batches	Age	26	53	11	56	33
	Age and size	0	31	16	37	10
	Age, size and sex	32	27	0	30	29
Breed	Large White x Landrace	8	62	0	51	19
	Large White x Landrace x Duroc	12	24	10	26	20
	Other	38	24	11	40	33
Artificial insemination	Yes	54	98	27	107	72
	No	4	0	0	4	0
Use own boars	Yes	44	55	10	86	23
	No	4	43	6	37	39
AI source	Own boars	12	11	16	18	21
	AI company	42	59	11	65	51
	Own boars and AI company	0	28	0	28	0

^a^ Refers to a batched farrowing system.

**Table 3:** Farm production, housing and disease herd management practices across 22 pig herds in southeastern Australia included in a survey of pre- and post-weaning scours.

		State			Farm size (sows)	
Variable	Category	VIC	NSW	SA	≤499	≥500
Farms		58	110	27	123	72
Farm production type	Indoor conventional	40	61	10	82	67
	Mixed (indoor and deep litter)	14	49	17	37	5
	Free range	4	0	0	4	0
Number of production sites	1	38	67	17	115	7
	≥ 2	20	29	10	8	51
Number of production buildings/Eco shelters	1–5	12	64	0	76	0
	≥ 6	46	46	27	47	72
Size farm (acres)	1–500	31	25	27	53	30
	≥ 501	17	74	0	60	31
Other livestock on farm	Yes	46	98	11	105	52
	No	12	12	16	18	20
Types of other livestock	Sheep	25	53	11	71	18
	Cattle	9	8	0	17	0
	Sheep and cattle	14	37	0	17	34
Crops	Yes	37	85	11	75	58
	No	19	25	16	46	14
Types of other crops	Wheat	0	16	0	16	0
	Wheat and another type of crop	10	51	0	36	25
	Other	27	18	11	23	33
Weaner feed type	Commercial (or makes own mix with commercial feed)	50	96	27	116	57
	Own mill	8	14	0	7	15
Weaner Feed Additives/Acids	Yes	43	59	21	77	46
	No	15	51	6	46	26
Water type	Bore/dam	31	58	0	49	40
	River/channel	17	14	0	9	22
	Town	0	12	6	18	0
	Combination	10	15	11	36	0
Antibiotics added to water	Yes	40	24	11	40	40
	No	18	86	16	83	32
Temperature control in weaner pens	Yes	51	88	10	96	54
	No	7	22	17	27	18
Heaters for temperature control in weaner pens	Yes	50	88	8	14	37
	No	8	22	19	109	35
Cooling system (thermo-regulated)/ sprinklers for temperature control in weaner pens	Yes	30	21	0	94	54
	No	28	89	27	29	18

The majority of farms bred Landrace or Landrace x Large White pigs. Most farms were greater than 500 acres and the majority housed pigs in indoor conventional sheds, having six or more buildings or Eco shelters on one site. Most weaner pens were within conventional shed systems, although each farm was slightly different, with the most common flooring being inorganic such as concrete and plastic slatting. Most weaner pens did not contain bedding, (organic material such as barley, straw, sawdust, ricehulls) however straw with the addition of another type of bedding was commonly used in Eco shelters.

Most sheds were more than 10 years old and open in design, most farms provided heaters as a source of temperature regulation to keep the weaners warm, but were less likely to provide a cooling source. The majority of farms followed the all in/all out production flow system, where all animals are removed from the accommodation, it is then cleaned and allowed to dry before new stock is moved into the space. The average weaning age ranged from 22 to 28 days and weaners were most commonly housed in medium sized groups of 16 to 199 piglets per pen/Eco shelter, based on age. The majority of farms have a cleaning protocol, 37% of these farms use pressure hosing with the addition of disinfectant to clean pens between batches of pigs.

The most common weaner feed type was purchased from a diverse range of commercial suppliers (89%) versus owning a mill and making their own feed (11%). Most farms supplemented weaner diet with feed additives, such as acids, spray-dried blood plasma or whey from milk by-products. Generally water was supplied from bores and dams. A large proportion of farms (40%) routinely treated each batch of piglets with antibiotics added to the water at weaning, with amoxicillin the most common antibiotic choice.

Pre- and post-weaning diarrhoea was constantly (or occasionally, within the last 12 months) experienced by most batches of pigs across the majority of farms. Nearly every farm (20 of 22 herds) had a vaccination program but half of the farms had experienced a recent disease outbreak within the last 12 months (the type and frequency of disease outbreaks varied between farms).

### Risk factors

The presence of diarrhoea was assessed at a pen level. We determined that of the 60 variables collected, the data show 6 variables were associated with pen diarrhoea status (p<0.2). Only ≤5% of the data are missing and thus were available for inclusion in a multivariate model (Table 4). Weaner status (pre-, post-weaned) was included (p = 0.275) as a potential confounding variable.

**Table 4:** Results of univariate risk factor analysis for presence of diarrhoea at a pen level in a survey of 22 pig herds in southeastern Australia.

Variable	Category	No diarrhoea	Diarrhoea	P-value
Weaned	No	77	16	0.275
	Yes	78	24	
Recent disease events	No	92	10	0.015
	Yes	63	30	
Post-weaning scours history	No	51	5	0.171
	Yes	104	35	
Infeed additives	No	50	22	0.184
	Yes	105	18	
In-water antibiotics	No	98	17	0.183
	Yes	57	23	
Bedding	No	107	15	0.037
	Yes	48	25	
Temperature control	No	46	2	0.028
	Yes	109	38	

A forwards stepwise regression using the likelihood ratio to select variables (p<0.05) identified the best-fitting model (-2 log likelihood 174.3, Nagelkerke R^2^ 0.179) to include recent disease and presence of bedding factors. Estimates of odds ratios were not confounded by weaner status (post-weaned vs. pre-weaned; <10% change in OR estimates) and the interaction between recent disease and bedding was statistically different. This model is shown in Table 5.

**Table 5:** Results of multivariate risk factor analysis for presence of diarrhoea at the pen level in a survey of 22 pig herds in southeastern Australia.

Term	Coefficient	SE	P-value	Odds Ratio	Lower 95% CI	Upper 95% CI
Intercept	-2.496	0.364	< 0.001	–	–	–
Recent disease						
No	0	–	–	1	–	–
Yes	1.183	0.419	0.005	4.381	0.362	2.005
Bedding						
No	0	–	–	1	–	–
Yes	0.963	0.391	0.014	3.715	0.197	1.729

### Bacteriology

The causal agents of diarrhoea were assessed in further detail at an individual sample level. During the survey, a total of 1,105 faecal samples were collected from 195 pens (93 pre- and 102 post-weaned) across 22 pig herds. Every herd had at least one β-haemolytic *E*. *coli* isolated from one out of the 50 faecal samples collected (S4 File). The prevalence of diarrhoea in pre- and post-weaned piglet samples was 32% and 47%, respectively. The prevalence of diarrhoea in pre-weaned pens was 17%, compared to 24% in post-weaned pens. The mean and median farm-level prevalence of pre- and post-weaned pen diarrhoea were 39% and 29%, respectively, and it ranged from 0 to 100%.

There was a significant difference (p = 0.0001) in the estimated prevalence of β-haemolytic *E*. *coli* between pre- and post-weaned piglet samples, 13% and 47%, respectively. β-haemolytic *E*. *coli* were present in 45% of diarrhea samples from 36 pens, (7 pre- and 29 post-weaned pens). The findings show that ETEC containing both an adhesin and an enterotoxin gene (capable of causing diarrhea) were more likely to contain both F4 and STb genes (present in 10 of 22 herds, Tables 6 and 7). However, the most prevalent single ETEC adhesin gene in β-haemolytic *E*. *coli* was F18 (32%) and the most prevalent enterotoxin gene was STb (30%), in 15 herds (Table 6). We also observed a higher association of β-haemolysis and F4:STb with diarrhoea samples than those containing F18:STb.

**Table 6:** ETEC virulence genes present in β-haemolytic *E*. *coli* isolates (n = 392) from a survey of 22 pig herds in southeastern Australia.

	Pre-weaned		Post-weaned	
Virulence attribute	Prevalence	Diarrhoea associated	Prevalence	Diarrhoea associated
F4	11	6	98	33
F18	14	0	113	21
F6	11	4	14	6
F5	0	0	27	0
F41	0	0	0	0
STa	5	1	95	20
STb	12	4	106	36
LT	14	7	62	31
Stx2e	0	0	10	2

**Table 7:** Farm presence of ETEC virulence genes present in β-haemolytic *E*. *coli* isolates (n = 22) in a survey of 22 pig herds in southeastern Australia.

Virulence attribute	Pre-weaned	Post-weaned
F4	4	11
F18	4	14
F6	1	3
F5	0	2
F41	0	0
STa	2	9
STb	4	15
LT	2	8
Stx2e	0	3

## Discussion

This study assessed 60 potential factors that could increase the risk of *E*. *coli* disease. In addition the prevalence of β-haemolytic ETEC *E*. *coli* in southeastern Australian pig herds was estimated. Only two key factors–recent disease events within the whole herd in the last 12 months and the presence of bedding, were significantly associated with the presence of diarrhoea in pens when a multiple logistical regression analysis was applied. A multitude of herd management practices could potentially impact recent disease events, including current disease status, poor infection control, low immunity within the herd, secondary infections, biosecurity, housing and reproductive practices, as well as a range of potentially unidentified environmental factors but these did not emerge in the most rigorous analysis, perhaps due to unmeasured confounders or insufficient statistical power. Furthermore, the continuous transfer of *E*. *coli* via faeces into the pen environment helps to maintain a stable bacterial population [16], contributing to the re-occurrence of disease events. Buddle *et al*. [6] found that applying the Madec and Josse [5] risk factor analysis to Australian conditions accurately reflected risk factors impacting piglets in the Australian environment but suggested that other risk factors were yet to be identified. Due to time and financial constraints, this study design could not include repeated measurements as performed by Buddle *et al*. [6] and Skirrow *et al*. [7] in order to determine if risk scores had increased or decreased. Additional risk factor variables from each farm were able to be recorded in order to include previously unidentified risk factors.

The results from this study suggest the presence of bedding significantly contributed to increasing the risk of *E*. *coli* disease in post-weaned piglets. The majority of herds in this study did not have bedding in weaner pens but, if used, the most popular bedding was straw. *E*. *coli* is commonly used as an indicator organism for environmental faecal contamination [17], but *E*. *coli* survival in straw is unstudied. If *E*. *coli* can withstand low nutrient availability, wide temperature fluctuations and competition from other microflora and there is a constant supply of carbon, growth outside its primary host is possible [17, 18]. Therefore, *E*. *coli* could survive in faeces within an indoor pig pen, further circulating within the environment with the potential to cause disease in young pigs as a result of coprophagic or burrowing behaviours [19].

The provision of bedding has many perceived benefits, such as providing a source of heat, providing stimuli for piglets, minimising dust and absorbing faecal matter [20–22]. Generally bedding is changed at the end of each batch. Our results suggest that bedding could potentially be harbouring pathogenic bacteria and is not changed as often as required in order to reduce the incidence of disease. Previous studies have outlined some disadvantages of using straw, such as increased costs associated with labour, hygiene and incompatibility with manure drainage systems [21, 23]. The estimated cost of production by implementing changes to reduce *E*. *coli* risk factors based on a small pig herd could potentially save producers $1.50 per pig in non-feed costs, including increased bedding and labour expenses but decreased veterinary and medical costs. However, this saving would vary depending on the number of breeding sows, feed cost and performance of the herd for example, feed conversion rate [24–26].

In this study, the prevalence of diarrhoea in pre- and post-weaned piglet pens in commercial herds in southeastern Australia was estimated to be 17% and 24%, respectively. This suggests that diarrhoea is an underlying problem across many herds and is likely a substantial cause of economic loss and animal welfare issues. Diarrhoea in piglets can be transient but, this study shows that persistent post-weaning diarrhoea within the last 12 months influences the presence of diarrhoea within pens.

In bivariate analysis, we observed a further four potential risk factors for piglet *E*. *coli* diarrhoea–temperature in weaner pens (thermostatically controlled versus no temperature control), a history of post-weaning scours (within the last 12 months), antibiotics added to the water supply and infeed additives (that may be the result of a causal relationship). The design of pig buildings varied between herds but the majority were open and exposed to temperature fluctuations and it was more common for buildings to have heating rather than cooling sources. Buddle *et al*. [6] suggested Australian pig buildings did not provide satisfactory environmental conditions for optimal pig health. These findings are supported in our study as it was found that absence of temperature control may be associated with the presence of diarrhoea in a pen–although this has not been reported in previous studies of pre-weaning [27] or post-weaning [7] diarrhoea.

When piglets are weaned, the transition from the sow’s colostrum antibody-rich milk to solid pelleted feed can have substantial impacts on the piglet’s gastrointestinal tract in terms of physiology, microbiology and immunology [28–30]. Additives, such as spray-dried blood plasma, may play a role in altering the intestinal structure in piglets, often reducing post-weaning diarrhoea, and are a suitable alternative to antibiotics [31, 32]. This study suggests that the administration of antibiotics in water and infeed additives play a substantial role in increasing the occurrence of diarrhoea, therefore antibiotic usage and infeed additive concentrations may need to be re-evaluated.

The presence of ETEC virulence genes is important for pathogenicity and clinical disease. For the purpose of this study, samples from both healthy and clinically-affected pens were screened for β-haemolytic *E*. *coli* to estimate the overall prevalence of pathogenic ETEC. It is common for healthy and clinically-infected piglets to show increased ETEC shedding after weaning [33] and for non-β-haemolytic *E*. *coli* to possess ETEC genes [14, 34] and may be due to numerous reasons such as the loss of virulence factors such as plasmid-encoded ST, resulting in a less virulent strain, or pig immunity due to maternal immunisation or prior infection. Our findings revealed the prevalence of β-haemolytic *E*. *coli* with a single ETEC gene versus both an adhesin and enterotoxin gene to be 70% (273/392) and 43% (167/273), respectively. This was slightly lower than ETEC prevalence levels reported in Queensland [13].

The enterotoxin gene Stx2e is commonly associated with edema disease. However, it has been recognised that some Shiga toxin producing *E*. *coli* (STEC) strains that possess Stx2e are capable of causing diarrhoea [4]. This study found Stx2e commonly occurred with the adhensin gene F18, which has been previously acknowledged in *E*. *coli* isolates from Australian piglets [34]. Although ETEC genes were present, unless they are being expressed they may have little impact on the piglets’ health. ETEC presence within the environment suggests that they have the potential to cause disease under suitable conditions. There are important implications for ETEC transmission from animals to humans, particularly since ETEC can also carry multi-drug resistance. Increased resistance levels have been shown in humans that come into direct contact with pigs such as abattoir workers [35] and presence of the same ESBL-producing *E*. *coli* in pigs and farmers from the same farm [36]. Further research is required to determine ETEC transmission from pigs to humans in an Australian context.

## Conclusions

Diarrhoea has significant detrimental impacts on piglets, however the findings of this study suggest by reducing the incidence of disease in a herd within the last 12 months and minimising the use of bedding (or maintaining fresh bedding) piglet and herd health can be increased. There are many positive associations with improved piglet health, such as reduced antibiotic use and veterinary bills and growth in profit margins and productivity. A consistently healthy herd will also assist in sustaining a high food safety standard with positive implications for human consumption.

## Supporting information

S1 File Questionnaire for recruiting participating Australian pig producers. (PDF)

S2 File Farm questionnaire on Australian pig herd management practices. (PDF)

S3 File Questionnaire data on Australian pig herd management practices. (XLSX)

S4 File ETEC virulence genes present in β-haemolytic *E*. *coli* isolated from Australian piglets. (XLSX)
