# Supplementary material for: Pre- and post-weaning scours in southeastern Australia: A survey of 22 commercial pig herds and characterisation of Escherichia coli isolates
Source: PLoS One. 2017 Mar 8;12(3):e0172528. doi: 10.1371/journal.pone.0172528 (PMC5342203; doi:10.1371/journal.pone.0172528)
Supplement: S1 File — (PDF) [file pone.0172528.s001.pdf]

S1 Appendix. Questionnaire for recruiting possible participating pig farms.

CONFIDENTIAL

Where is your farm located? ____

What type of farm production do you run? For example, intensive indoor or extensive outdoor? ____

How many sows do you have at the moment? ______________________________________

Is post-weaning scours a problem in your herd? ____________________________________

When was the last time you had an outbreak of post-weaning scours in your herd?

____

At what age do you wean your piglets? ____

How soon after weaning do you see diarrhoea? _____________________________________

Is the scours usually watery or bloody? ____

Do you lose any piglets to this disease? ____

What time of year do see post-weaning scours? ____________________________________

If I sampled sometime between August and October, would this suit you? _______________

If not, what time would you prefer? ____

What is your preferred method of contact? Phone/email? ______________________________
