# Supplementary material for: Pre- and post-weaning scours in southeastern Australia: A survey of 22 commercial pig herds and characterisation of Escherichia coli isolates
Source: PLoS One. 2017 Mar 8;12(3):e0172528. doi: 10.1371/journal.pone.0172528 (PMC5342203; doi:10.1371/journal.pone.0172528)
Supplement: S2 File — (PDF) [file pone.0172528.s002.pdf]

S2 Appendix. Farm questionnaire.

CONFIDENTIAL – Farm questionnaire

Farm Code: **Date:**

Location of farm?____

Breed?____

Average number of sows?____

Farm production type? (Free range/intensive indoor/semi-intensive/Eco shelter)____

Number of production sites/buildings or sheds?_______________________________

How many acres is your farm?____

Do you run other livestock/grow crops on the same property? If yes what are they?

____

How many pigs are in the farrowing shed/pens?________________________________

How many pigs are in the weaner shed?______________________________

Do you see scours during the suckling period?______________________________

Do you see scours during post-weaning?______________________________

List all vaccinations given to sows?______________________________

____

List all vaccinations given to weaners?______________________________

____

List all vaccinations given to pre-weaners?______________________________

Have you had any problems with diseases other than *E. coli* recently (within the last 12 months?______________________________

____

Do you have a closed herd? If yes, for how long?______________________________

Do you use AI? If yes, where is it sourced?______________________________

Farrowing crate set up:

Weaner pen set up:

Pen details?

Average size of weaner pens (number of piglets/pen)?________________________________

Bedding used in the weaner pens? Type?____

Weaner feed type? (Creep/acidified) ____

Who producers the feed? If supplied by a commercial company, name supplier?____

Water type? (dam/bore)____

Are any antibiotics added to the weaners water? If yes, what is added?__________________

____

Temperature control in weaner pens? If yes, what is used? (sprinklers/heat lamps):____

Average age of piglets at weaning?____

How are the weaners separated into groups? (same litter kept together/housed based on weight/size) ____

____

How often are the weaner pens cleaned?___________________________________

How do you get rid of your effluent?______________________________________

How many pigs do you slaughter each week?_______________________________

Which abattoir do you use?____

Do you use your own truck to transport pigs to the abattoir? If no, how are they transported?____
